# BMC *Endocrine Disorders* reviewer acknowledgement 2015

**DOI:** 10.1186/s12902-016-0091-0

**Published:** 2016-02-15

**Authors:** Tim Shipley

**Affiliations:** BioMed Central, Floor 6, 236 Gray’s Inn Road, London, WC1X 8HB UK

## Abstract

The editors of BMC Endocrine Disorders would like to thank all our reviewers who have contributed to the journal in Volume 15 (2015).

**Martina Rossi**

Italy

**Dimitra Argyro Vassiliadi**

Greece

**Janaki Parajuli**

Nepal

**Daya Ram Pokharel**

Nepal

**Marian Benroubi**

Greece

**Nikolaos Papanas**

Greece

**Magnus R. Dias-Da-Silva**

Brazil

**Steven Levine**

USA

**Eoin Noctor**

Denmark

**Yeyi Zhu**

USA

**Claudio Letizia**

Italy

**Vincenzo Salpietro**

Italy

**Romulo Dezonne**

Brazil

**Alfredo Pontecorvi**

Italy

**Jonathan Bogan**

USA

**Altan Onat**

Turkey

**Sobia Ali**

Pakistan

**Rayaz Malik**

Qatar

**Cristina Capatina**

Romania

**Fatih Tanriverdi**

Turkey

**Philip Haycock**

UK

**Nicholas Tentolouris**

Greece

**Vsiliki Benetou**

Greece

**Lindsay Jaacks**

USA

**Giovanni Corona**

Italy

**Paresh Dandona**

USA

**Daniele Santi**

Italy

**Rachel Crowley**

Ireland

**Fahrettin Kelestimur**

Turkey

**Tommy Kyaw-Tun**

Ireland

**Francesco Ferrau**

Italy

**Hidenori Fukuoka**

Japan

**Christinna Olesen**

Denmark

**Andrew Grandinetti**

USA

**Elizabeth Beverly**

USA

**Ching-Ju Chiu**

Taiwan

**Neil Gittoes**

UK

**Fevziye Kabukcuoglu**

Turkey

**Thomas Vizhalil Paul**

India

**Yoshifumi Saisho**

Japan

**Nagaaki Tanaka**

Japan

**Michael O'Reilly**

UK

**Tríona O'Shea**

Ireland

**Bala Bhagavath**

USA

**Giacomo Tirabassi**

Italy

**Michiharu Komatsu**

Japan

**Farah Mccrate**

Canada

**Georgios Valsamakis**

Greece

**Sarita Bajaj**

India

**Jessica Kiefte-De Jong**

Netherlands

**Raffaele Ciampi**

Italy

**Malgorzata Oczko-Wojciechowska**

Poland

**Nihal Hatipoglu**

Turkey

**Jan Stepan**

Czech Republic

**Milan Piya**

UK

**Florian Seyfried**

Germany

**Simon Lebech Cichosz**

Denmark

**Laercio Franco**

Brazil

**Kevin Brumund**

USA

**Andrea Frasoldati**

Italy

**Fabio Fernandes-Rosa**

France

**Antonio Lerario**

Brazil

**Davide Carvalho**

Portugal

**Pm Clark**

UK

**Carole Spencer**

USA

**Fabrice Gouilleux**

France

**Ciara Mcdonnell**

Ireland

**Sandra Zinkel**

USA

**Erica Gentilin**

Italy

**Ulrich Renner**

Germany

**C S Bal**

India

**Bingyin Shi**

China

**Marco Gallo**

Italy

**Ulrike Rothe**

Germany

**Sijun Dong**

China

**Brigitte Le Magueresse-Battistoni**

France

**Ambika Ashraf**

USA

**Elizabeth Ramos Lopez**

Germany

**Ohk-Hyun Ryu**

South Korea

**Jane Burns**

USA

**Fanny Rancière**

France

**Iwao Sugitani**

Japan

**Jim Yeung**

USA

**Isabel Huguet**

Spain

**Zuleyha Karaca**

Turkey

**Andreas Muth**

Sweden

**Mihail Zilbermint**

USA

**Naim Maalouf**

USA

**Luigi Petramala**

Italy

**Francesco Tassone**

Italy

**Xiangbing Wang**

USA

**Carla Carvalho**

Brazil

**Junia Santos-Silva**

Brazil

**Serdar Güler**

Turkey

**William Tormey**

Ireland

**Laura Audi**

Spain

**Berit Kriström**

Sweden

**Mieczyslaw Szalecki**

Poland

**Hristina Denic**

USA

**Winda Liviya Ng**

Australia

**Gaztambide Sonia**

Spain

**Bin Wu**

China

**Jacqueline Cleator**

UK

**Nicolai Wewer Albrechtsen**

Denmark

**Tatsuya Kondo**

Japan

**Giorgio Lelli**

Italy

**Mu Chen**

USA

**Goodarz Danaei**

USA

**Danxia Yu**

USA

**Alexander Kokkinos**

Greece

**Stelios Tigas**

Greece

**Gerhard Binder**

Germany

**Tsung-Hsueh Lu**

Taiwan

**Athanasia Papazafiropoulou**

Greece

**Kirsten Rennie**

UK

**Oktay Bilgir**

Turkey

**Jorge Pacheco-Rosado**

Mexico

**Ioannis Ioannidis**

Greece

**Stavros Liatis**

Greece

**Carl Lombard**

South Africa

**Mark Cooper**

Australia

**María Isabel Sevilla**

Spain

**Hui-Jen Tsai**

Taiwan

**Run Yu**

USA

**Sabrina Corbetta**

Italy

**Laura Gianotti**

Italy

**Katherine Valeros**

Singapore

**Gesine Meyer**

Germany

**Lisanne Van Der Plas-Smans**

Netherlands

**Joanna Stewart**

New Zealand

**Giulia Rastrelli**

Italy

**Bart Clarke**

USA

**Masahito Ogura**

Japan

**Katharine Barnard**

UK

**Cephas Sialubanje**

Zambia

**David Drozek**

USA

**Lillian Kent**

Australia

**Louise Maple-Brown**

Australia

**Tony Huynh**

Australia

**Maria New**

USA

**Richard Ross**

UK

**Jean Joel Bigna**

Cameroon

**José Rosa Neto**

Brazil

**Claudio Zoppi**

Brazil

**Nigel Irwin**

UK

**Krzysztof Safranow**

Poland

**Samir Assaad Khalil**

Egypt

**Enzo Sella**

USA

**Martin Cuesta**

Spain

**Diarmuid Smith**

Ireland

**Bjorn Dahlback**

Sweden

**Eric Sijbrands**

Netherlands

**Ching-Chu Chen**

Taiwan

**Vaia Lambadiari**

Greece

**Konstantinos Makrilakis**

Greece

**Monika Buraczynska**

Poland

**Parimala Narne**

India

**Daniel Petrovic**

Slovenia

**Decio Armanini**

Italy

**Daniel Gonzalez Maglio**

Argentina

**Theresa Allain**

Malawi

**William B. Grant**

USA

**Andre Renzaho**

Australia

**Karsten Müssig**

Germany

**Diana Hernández-Romero**

Spain

**Arndt Buessing**

Germany

**Konstantinos Markakis**

UK

**Toralf Melsom**

Norway

**Maria Lucia Correa-Giannella**

Brazil

**Emerielle Vanzela**

Brazil

**Iftikhar Ahmed**

Pakistan

**Rosario Hirata**

Brazil

**Muhammad Zafar Hydrie**

Pakistan

**Xiaoyue Pan**

USA

**Edmund Rucker**

USA

**Jennifer Teske**

USA

**Guangrui Yang**

USA

**Deborah Chyun**

USA

**Kristian Karstoft**

Denmark

**Birger Thorsteinsson**

Denmark

**Francis Finucane**

Ireland

**Evangelos Rizos**

Greece

**Yoshitaka Hayashi**

Japan

**Margaret Keil**

USA

**Martin Bidlingmaier**

Germany

**Carla Bizzarri**

Italy

**Assunta Albanese**

UK

**Musarrat Riaz**

Pakistan

**Yoshihito Fujita**

Japan

**Damian Romero**

USA

**Flavia Prodam**

Italy

**Giovanna Muscogiuri**

Italy

**Angela Leung**

USA

**Daniel Bichet**

Canada

**Joseph Verbalis**

USA

**Sai-Ching Yeung**

USA

**Diana Learoyd**

Australia

**Trayambak Basak**

USA

**Emanuela Ricciotti**

USA

**Ji-Houn Kang**

Republic Of Korea

**Peter Igaz**

Hungary

**Azizi Seixas**

USA

**Ellen Toth**

Canada

**Harish Ranjani**

India

**M Druce**

UK

**Mark Gurnell**

UK

**Malgorzata Wiench**

UK

**Taiga Shibata**

Japan

**Kazuhiko Sakaguchi**

Japan

**Takashi Nomiyama**

Japan

**Gustavo Santos**

Brazil

**Fernando Rodrigues Morais Abdulkader**

Brazil

**Chaoxing Yang**

USA

**George Coura-Filho**

Brazil

**Mark Hannon**

UK

**Jun-Yen Yeh**

USA

**Elzbieta Petriczko**

Poland

**Ana M Ramos-Levi**

Spain

**David Giovannucci**

USA

**Robert Roses**

USA

**Jochen Schneider**

Luxembourg

**Symeon Tournis**

Greece

**Tine Willum Hansen**

Denmark

**Shizukiyo Ishikawa**

Japan

**Maddalena Trombetta**

Italy

**Bee Tan**

UK

**Leanne Hodson**

UK

**Dimitra Vassiliadi**

UK

**Jessica Ares**

Spain

**Ana Carolina Santos De Souza Galvão**

Brazil

**Akira Kubota**

Japan

**Toshio Iwakura**

Japan

**Akinobu Nakamura**

Japan

**Claire Higham**

UK

**Nigel Glynn**

Ireland

**Vasilis Chortis**

UK

**Giacomo Sturniolo**

Italy

**Shuanzeng Wei**

USA

**Carla Moran**

UK

**Krishna Chatterjee**

UK

**Amin Talebi Bezmin Abadi**

Islamic Republic Of Iran

**Peter Schwarz**

Germany

**Ulla Feldt-Rasmussen**

Denmark

**John Doupis**

Greece

**Chrysi Koliaki**

Greece

**Alexandra Bargiota**

Greece

**Marinela Tzanela**

USA

**Rohana Ghani**

Malaysia

**Rajaventhan Srirajaskanthan**

UK

**Davi' Maria Vittoria**

Italy

**Jonathan Hazlehurst**

UK

**Jeremy Turner**

UK

**Ioanna Eleftheriadou**

Greece

**Harvey Chiu**

USA

